# Associations of Job Stress Indicators with Oxidative Biomarkers in Japanese Men and Women

**DOI:** 10.3390/ijerph10126662

**Published:** 2013-12-02

**Authors:** Jiro Takaki

**Affiliations:** Department of Public Health and Occupational Medicine, Graduate School of Medicine, Mie University, 2-174 Edobashi, Tsu, Mie 514-8507, Japan; E-Mail: jirosinryounaika-tky@umin.ac.jp; Tel.: +81-59-232-1111 (ext. 6372); Fax: +81-59-231-5012.

**Keywords:** biomarkers, demands-control-support model, effort-reward imbalance model, oxidative stress, occupational health, psychological stress, sex factors

## Abstract

Some researchers have suggested that oxidative damage may be one of the mechanisms linking job stress with coronary heart disease. The aim of this study was to investigate the association between job stress indicators and oxidative biomarkers. The study included 567 subjects (272 men, 295 women) who answered questionnaires related to their work and underwent a medical examination. Job stress evaluated using the demands-control-support model was measured using the Job Content Questionnaire. Effort-reward imbalance was measured using the Effort-Reward Imbalance Questionnaire. Urinary hydrogen peroxide (H_2_O_2_) and 8-hydroxy-2'-deoxyguanosine (8-OHdG) were measured by the modified ferrous ion oxidation xylenol orange version-1 method and enzyme-linked immunosorbent assay, respectively. In men, the changes in the odds ratios for high urinary H_2_O_2_ associated with a 1-standard-deviation (SD) increase in worksite social support were 0.69 (95% confidence interval (CI) 0.53, 0.91) univariately and 0.68 (95%CI 0.51, 0.90) after adjustment for covariates. The change in the odds ratio for high urinary H_2_O_2_ associated with a 1-SD increase in effort-reward ratio was 1.35 (95% CI 1.03, 1.78) after adjustment for covariates. In women, there were no significant associations of the two job stress indicators with urinary H_2_O_2_ and 8-OHdG levels after adjustment for covariates (*p* > 0.05).

## 1. Introduction

Several studies have suggested that psychological stress induces production of reactive oxygen species (ROS) [1,2,3,4,5,6,7,8,9]. Job stress has been recognized as one of the major risk factors for coronary heart disease (CHD) [2,10]. Oxidative damage has also been identified as a major risk factor for CHD, and increased vessel wall oxidative stress is a pathogenic feature of atherosclerosis and hypertension [11]. Thus, some researchers suggest that oxidative damage may be one of mechanisms linking job stress with CHD [1,2]. In rats, levels of 8-hydroxy-2'-deoxyguanosine (8-OHdG), an oxidation product of nuclear DNA bases, increased in liver after exposure to psychological stress [3]. Immobilization-induced stress, a combination of psychological and psycho-physical stress, was reported to cause oxidative damage to lipids, proteins, and DNA in the rat brain [4]. In a study in humans, chronic and subchronic psychological stress were associated with increased oxidative stress evaluated using plasma superoxide anion (O_2_^−^) and malondialdehyde levels [5]. University students under examination stress had increased oxidative stress as shown by oxidative damage to DNA and sensitivity to lipid oxidation [6]. Psychological stress, delivering a speech, and self-reported stress were associated with increased bilirubin oxidative metabolites [7]. Perceived workload and perceived stress, which were each evaluated using one simple question were associated with increased urinary 8-OHdG levels in female workers, but not in male workers [8]. Job stress evaluated using the job demands/control ratio was associated with urinary 8-OHdG levels in women, but not in men [2]. In contrast, job stress evaluated using the effort-reward imbalance model was not associated with biopyrrin, a bilirubin oxidative metabolite [9], nor urinary 8-OHdG [2] levels. 

In this study, job stress was evaluated using the demand-control-support model [12] and the effort-reward imbalance model [13], and their associations with urinary 8-OHdG levels were determined. The associations between job stress indicators and urinary hydrogen peroxide (H_2_O_2_), as a biomarker of ROS [14,15], were also investigated for the first time. Because urinary H_2_O_2_ levels are influenced by diet [14], measurements were made after overnight fast. As previous studies have suggested gender differences in the relationships between job stress and 8-OHdG levels [2,8], the data in men and women were analyzed separately.

## 2. Methods

### 2.1. Subjects

The subjects in this study were recruited from the full-time workers (n = 1,003) in four different industries in Japan. The purpose and procedures of the survey were explained to the participants in the documents. Written informed consent was obtained from all participants. Approximately 2 weeks after distribution of the self-administered questionnaires, they were returned by 605 workers (response rate: 60.3%) at their medical examination in 2007. Because of missing data, 567 workers (272 men, 295 women) were included in the analyses. This study was approved by the ethics committee of the Graduate School of Medicine, Dentistry and Pharmaceutical Sciences, Okayama University, and was performed according to the Declaration of Helsinki.

### 2.2. Parameters

#### 2.2.1. Job Stress Evaluated Using the Demands-Control-Support Model

Job stress evaluated using the demands-control-support model was measured with the Job Content Questionnaire (JCQ), developed by Karasek [12]. The JCQ includes scales for job demands (five items; score range, 12–48), job control (nine items; score range, 24–96), and worksite social support (eight items; score range, 8–32), with four-point response options from 1 (strongly disagree) to 4 (strongly agree). The JCQ was translated into Japanese and the internal consistency reliability and factor and construct validity have been reported to be acceptable [16]. The job strain index, which is calculated as job demands divided by job control, has been used as an indicator of job strain, with higher scores indicating greater strain [17]. 

#### 2.2.2. Job Stress Evaluated Using the Effort-Reward Imbalance Model

Job stress evaluated using the effort-reward imbalance model was measured with the Effort-Reward Imbalance Questionnaire (ERIQ), developed by Siegrist [13]. The ERIQ comprises two main scales—extrinsic effort (six items; score range 6–30) and reward (11 items; score range 11–55)—with a five-point response option. An effort-reward ratio, which is calculated as extrinsic effort divided by reward and multiplied by 6/11, has been used as an indicator of effort-reward imbalance, with higher scores indicating more stressful situations [18]. The ERIQ has been translated into Japanese, and its reliability and validity have been reported as acceptable [19]. 

#### 2.2.3. Urinary H_2_O_2_ and 8-OHdG

Spot urine samples were collected after overnight fasting for at least 10 hours during the subjects’ medical examination. The samples were stored at −80 °C until analysis. Urinary H_2_O_2_ was measured by the ferrous ion oxidation xylenol orange version-1 (FOX-1) method [20]. The FOX-1 assay was modified to be more specific to H_2_O_2_ by performing it in the presence and absence of catalase, the enzyme that significantly destroys H_2_O_2_ [20]. Urinary 8-OHdG was measured by an enzyme-linked immunosorbent assay (ELISA) kit from the Japan Institute for the Control of Aging (JaICA) [21]. Evans *et al.* reported that apparent improvement in the specificity of the JaICA ELISA kit brought mean liquid chromatography–tandem mass spectrometry (LC-MS/MS) and ELISA measurements of urinary 8-OHdG into agreement [22]. Based on the recommendation by Evans *et al.* [22] and Song *et al.* [23], the incubation with primary antibody was performed at 4 °C overnight for reduction of cross-reactions. A previous study indicated that the correlation coefficient of 8-OHdG measurements by ELISA between spot and 24-hour urine samples was 0.87 [24]. The intra- and inter-assay coefficients of variation were reliably <10% for both measurements of urinary H_2_O_2_ and 8-OHdG. Values for H_2_O_2_ and 8-OHdG were adjusted for urinary creatinine concentration. 

#### 2.2.4. Covariates

Age, body mass index (BMI), cigarette smoking, alcohol consumption, exercise, and total vegetable intake were included in the analyses as covariates. These factors are associated with ROS [1,25,26,27,28,29]. Age was calculated from the date of answering the questionnaires and the date of birth. BMI was calculated as body weight (kg) divided by the square of body height (m^2^), which were measured at the medical examination. The categories of cigarette smoking were: heavy smokers, smoking >20 pack years; moderate smokers, smoking 1 to 20 pack years; nonsmokers, ex-smokers, or occasional smokers other than moderate or heavy smokers. The categories of alcohol consumption were as follows: nondrinkers; those who drank alcohol only once per week or less; drinkers, who drank more than once per week. The categories of exercise were: less than once per week; once per week or more. Total vegetable intake (g/day) was measured by a validated food frequency questionnaire [30]. 

### 2.3. Statistical Analysis

Differences in continuous variables were compared between men and women using unpaired *t*-test. Categorical variables were compared using the chi-square test. The association between each psychosocial factor at work with each oxidative biomarker was assessed using univariate and multivariate logistic regression analyses. High urinary H_2_O_2_ or 8-OHdG was defined as upper quartile of urinary H_2_O_2_ or 8-OHdG in each gender, respectively. All *p*-values were two-tailed, with *p* < 0.05 considered statistically significant. All statistical analyses were performed using SPSS version 20 (IBM Japan, Chuo-ku, Tokyo, Japan).

## 3. Results

Participant characteristics according to gender are shown in Table 1. Age, BMI, cigarette smoking, alcohol consumption, exercise, total vegetable intake, job control, worksite social support, job strain, reward, and effort-reward ratio were significantly different between men and women. 

Associations of each psychosocial factor at work with urinary H_2_O_2_ and 8-OHdG are shown in Table 2. In men, worksite social support was significantly negatively associated with urinary H_2_O_2_ both in univariate analysis and after adjustment for covariates. The effort-reward ratio was significantly positively associated with urinary H_2_O_2_ only after adjustment for covariates. In women, worksite social support was significantly negatively associated with urinary 8-OHdG in univariate analysis, but did not remain significant after adjustment for covariates. There were no other significant associations with urinary H_2_O_2_ nor 8-OHdG levels.

## 4. Discussion

In men, worksite social support was negatively associated with urinary H_2_O_2_ before and after adjustment for covariates. Effort-reward ratio was positively associated with urinary H_2_O_2_ only after adjustment for covariates. In women, no associations between the two job stress models and H_2_O_2_ were found. To date, no study has tested the association between the job stress indicators in the two models and H_2_O_2_.

**Table 1:** Participant characteristics according to gender.

Characteristic	Men (n = 272)			Women (n = 295)			*p* ^a^
	Mean	SD	Range	Mean	SD	Range	
Age (years)	43.5	10.0	20.0–67.7	40.3	10.6	18.6–65.4	<0.001
BMI (kg/m^2^)	23.7	3.5	16.1–37.2	21.7	3.6	14.5–39.7	<0.001
Total vegetable intake (g/day)	124.8	80.2	0–645	144.4	93.2	4–557	0.007
Urinary H_2_O_2_ (μmol/g creatinine)	5.54	6.83	0.01–51.38	6.32	11.13	0.01–101.45	0.309
Urinary 8-OHdG (μg/g creatinine)	8.86	3.36	2.13–21.87	9.25	4.03	0.05–25.56	0.216
*Job Content Questionnaire*							
Job demands	32.1	5.5	12–48	32.4	5.6	12–48	0.455
Job control	65.7	10.4	24–90	62.2	10.8	24–90	<0.001
Worksite social support	22.3	3.2	12–32	21.7	4.0	8–32	0.044
Job strain index **^b^**	0.50	0.10	0.24–0.95	0.54	0.15	0.21–1.42	<0.001
*Effort-Reward Imbalance Questionnaire*							
Extrinsic effort	13.3	4.2	6–29	13.5	4.7	6–28	0.660
Reward	44.3	6.8	21–55	42.2	8.2	13–55	0.001
Effort-reward ratio	0.58	0.28	0.22–2.10	0.64	0.37	0.20–2.44	0.029
	**n**	**%**		**n**	**%**		
Cigarette smoking							<0.001
Moderate smoker **^c^**	85	31.3		40	13.6		
Heavy smoker **^d^**	80	29.4		6	2.0		
Alcohol consumption							<0.001
Once per week or less, but not none	64	23.5		127	43.1		
More than once per week	142	52.2		50	16.9		
Exercising once per week or more	104	38.2		61	20.7		<0.001

Notes: SD = standard deviation, BMI = body mass index, H_2_O_2_ = hydrogen peroxide, 8-OHdG = 8-hydroxy-2'-deoxyguanosine; **^a^** Continuous variables were compared using the unpaired *t*-test, and categorical variables were compared using the chi-square test; ^**b**^ Calculated as job demands divided by job control; ^**c**^ Current smokers with a smoking history of 1 to 20 pack years; ^**d**^ Current smokers with a smoking history of >20 pack years.

As for urinary 8-OHdG, no association was found in either men or women between the job stress indicators and 8-OHdG after adjustment for covariates. 

In human studies, H_2_O_2_ was evaluated as a biomarker of ROS [14,15] and showed high values in cancer patients [31], and associations with 8-OHdG, total cholesterol, and insulin dependency in healthy subjects [32]. H_2_O_2_ is a major contributor to tissue injury after transient ischemia in a variety of organs including the heart [33,34]. In this study, an association between effort-reward ratio and urinary H_2_O_2_ were found in men, but not in women. This is consistent with previous studies which failed to find an association of job stress with CHD in women or found an association which was weaker in women than in men [10,35,36,37]. Oxidative damage may partly mediate job stress and CHD as some researchers have suggested [1,2]. The gender difference could also be explained in terms of gender roles [10,37]. Threats to work-related social status are most stressful to males as they endanger their core traditional social identity, whereas women, in general, have alternative options for securing their social identity. Moreover, as pointed out earlier, an adverse psychosocial environment outside work, the home, may elicit more stressful reactions in women than the work environment [10,37]. 

**Table 2:** Changes in the odds ratio associated with a 1-SD increase in the psychosocial factors at work.

Factor	Men					Women			
	Univariate Model			Adjusted Model ^a^		Univariate Model		Adjusted Model ^a^	
Odds ratio for high **^b^** urinary H_2_O_2_ (95% confidence interval)									
Job Content Questionnaire									
Job strain index **^c^**	0.96	(0.77, 1.33)	1.07		(0.80, 1.43)	0.97	(0.74, 1.27)	1.00	(0.76, 1.32)
Worksite social support	**0.69**	**(0.53, 0.91)**	**0.68**		**(0.51, 0.90)**	0.98	(0.75, 1.28)	1.05	(0.79, 1.41)
Effort-Reward Imbalance Questionnaire									
Effort-reward ratio	1.26	(0.97, 1.63)	**1.35**		**(1.03, 1.78)**	1.02	(0.79, 1.33)	1.05	(0.79, 1.40)
Odds ratio for high **^b^** urinary 8-OHdG (95% confidence interval)									
Job Content Questionnaire									
Job strain index **^c^**	0.98	(0.74, 1.29)	1.02		(0.76, 1.37)	0.92	(0.70, 1.22)	0.96	(0.71, 1.30)
Worksite social support	0.98	(0.75, 1.29)	1.00		(0.75, 1.33)	**0.75**	**(0.57, 0.98)**	0.87	(0.65, 1.17)
Effort-Reward Imbalance Questionnaire									
Effort-reward ratio	1.10	(0.84, 1.43)	1.14		(0.86, 1.51)	1.14	(0.88, 1.46)	1.04	(0.79, 1.38)

Notes: SD = standard deviation, H_2_O_2_ = hydrogen peroxide, 8-OHdG = 8-hydroxy-2'-deoxyguanosine; ^**a**^ Adjusted for age, body mass index, cigarette smoking, alcohol consumption, exercise, and total vegetable intake; ^**b**^ Upper quartile; ^**c**^ Calculated as job demands divided by job control; Bold values signify statistical significance.

ELISA measurements of urinary 8-OHdG have been questioned by several scientists [38,39,40,41,42]. Cooke *et al*. reported discrepancy between salivary 8-OHdG values evaluated using LC-MS/MS and ELISA [40] and indicated that ELISA approaches continued to overestimate 8-OHdG levels and were not sufficiently specific for accurate quantification [41]. European Standards Committee on Urinary (DNA) Lesion Analysis and their colleagues reported that ELISA measurements of 8-OHdG showed more within-technique variation than did the chromatographic techniques and, for the urine samples, reported higher values [42]. Though Evans *et al.* reported improvement in the specificity of the JaICA ELISA kit used in this study, chromatographic techniques still remain the gold standard techniques for analysis of urinary 8-OHdG [22]. In a previous study, urinary 8-OHdG measured using ELISA was positively associated with the job demands/control ratio only in women and only after adjustment for various covariates, and was not associated with the effort-reward ratio in either gender [2]. This was in contrast to the present study. The present results must be confirmed in further studies of the associations between job stress indicators and 8-OHdG values evaluated using LC-MS/MS.

This study has some limitations. First, because this study used a cross-sectional design, it was difficult to determine the causal nature of the observed relationships. Longitudinal research is necessary to clarify causality. Second, because this study used convenience sampling, the results may not be applicable to the entire workforce. However, because subjects were recruited from four entirely different industries and the response rate was more than 50%, some generalizability can be expected. Third, unknown factors may confound the findings, though measurements were made after an overnight fast, and age, obesity, cigarette smoking, alcohol consumption, exercise, and total vegetable intake were adjusted for. 

## 5. Conclusions

The study found that, in men, worksite social support was negatively associated with urinary H_2_O_2_ before and after adjustment for covariates, and the effort-reward ratio was positively associated with urinary H_2_O_2_ after adjustment for covariates. In women, there were no significant associations between the indicators of job stress and oxidative biomarkers after adjustment for covariates. These results may contribute to the clarification of the mechanism of the effect of psychological stress on the body.
